# Multidrug-Resistant *Shigella dysenteriae* Type 1: Forerunners of a New Epidemic Strain in Eastern India?

**DOI:** 10.3201/eid0903.020352

**Published:** 2003-03

**Authors:** Dipika Sur, Swapan K. Niyogi, Shravani Sur, Kamal K. Datta, Yoshifumi Takeda, Gopinath Balakrish Nair, Sujit K. Bhattacharya

**Affiliations:** * National Institute of Cholera and Enteric Diseases, Kolkata, India; † Burdwan Medical College, Burdwan, West Bengal; ‡ Jissen Women’s University, Tokyo, Japan; § International Centre for Diarrhoeal Diseases Research, Dhaka, Bangladesh

**To the Editor:** Multidrug-resistant *Shigella dysenteriae* type 1 caused an extensive epidemic of shigellosis in eastern India in 1984 (*1*). These strains were, however, sensitive to nalidixic acid, and clinicians found excellent results by using it to treat bacillary dysentery cases. Subsequently, in 1988 in Tripura, an eastern Indian state, a similar outbreak of shigellosis occurred in which the isolated strains of *S. dysenteriae* type 1 were even resistant to nalidixic acid (*2*). Since then, few cases of shigellosis have occurred in this region, and *S. dysenteriae* type 1 strains are scarcely encountered (*3*). In other regions of the world, especially in Southeast Asia, low-level resistance to fluoroquinolones in S*higella* spp. has been observed for some time (*4*,*5*).

After a lapse of almost 14 years, clusters of patients with acute bacillary dysentery were seen at the subdivisional hospital, Diamond Harbour, in eastern India. No cases of dysentery had been reported during the comparable period in previous years. A total of 1,124 case-patients were admitted from March through June 2002. The startling feature of these infections was their unresponsiveness to even the newer fluoroquinolones such as norfloxacin and ciprofloxacin, the drugs often used to treat shigellosis. Clinicians tried various antibiotics, mostly in combinations, without benefit. Clinicians also randomly used anti-amoebic drugs without success.

An investigating team collected nine fresh fecal samples from dysentery patients admitted to this hospital; 4 (44%) yielded *S. dysenteriae* type 1 on culture. For isolation of *Shigella* spp., stool samples were inoculated into MacConkey agar and Hektoen Enteric agar (Difco, Detroit, MI), and the characteristic colonies were identified by standard biochemical methods (*6*). Subsequently, serogroups and serotypes were determined by visual inspection of slide agglutination tests with commercial antisera (Denka Seiken, Tokyo). Antimicrobial susceptibility testing was performed by an agar diffusion disk method, as recommended by the National Committee for Clinical Laboratory Standards (*7*). Results showed that the organisms were resistant to all commonly used antibiotics, including the fluoroquinolones (norfloxacin and ciprofloxacin) but were sensitive to ofloxacin. On our advice, the clinicians used ofloxacin with good results.

A similar outbreak of *S. dysenteriae* type 1 occurred in the northern part of West Bengal in eastern India among tea garden laborers from April 2002 to May 2002; 1,728 persons were affected (attack rate of 25.6%). Sixteen persons died. The isolated *S. dysenteriae* type 1 strains were found intermediately sensitive to fluroquinolones with an MIC of 2 μg/mL (K. Sarkar, S. Ghosh, S.K. Niyogi, S.K. Bhattacharya, pers. commun.).

This drug-resistant Shiga bacillus is highly likely to spread further and will certainly pose a major therapeutic challenge unless adequate preventive measures are immediately instituted to contain its spread. Appropriate awareness programs for the community and reorientation training for physicians and other health personnel would be helpful to prevent further transmission of these resistant organisms. Alternative drugs to treat drug-resistant cases and an effective vaccine are also needed.
